# Bivalent circular RNA vaccines against porcine epidemic diarrhea virus and transmissible gastroenteritis virus

**DOI:** 10.3389/fimmu.2025.1562865

**Published:** 2025-03-31

**Authors:** Weibing Zhang, Lei Wang, Liyu Chu, Xu Ma, Wenjing Gao, Yarong Wu, Yongfeng Qiao, Xianjun Wang, Lu Zhao, Hong Hu, Xiaoyu Li, Ding Zhang, Tao Song, Guocan Yu, Haidong Wang, Chunbo Dong, Zhida Liu

**Affiliations:** ^1^ College of Veterinary Medicine, Shanxi Agricultural University, Jinzhong, China; ^2^ Shanxi Academy of Advanced Research and Innovation, Taiyuan, China; ^3^ Department of Biochemistry and Molecular Biology, College of Basic Medical Sciences, Shanxi Medical University, Taiyuan, China; ^4^ Hebei Key Laboratory of Preventive Veterinary, College of Animal Science and Technology, Hebei Normal University of Science and Technology, Qinhuangdao, China; ^5^ Ankerui (Shanxi) Biological Cell Co., Ltd., Taiyuan, China; ^6^ Key Laboratory of Bioorganic Phosphorus Chemistry & Chemical Biology, Department of Chemistry, Tsinghua University, Beijing, China

**Keywords:** circRNA vaccine, bivalent, porcine epidemic diarrhea virus, transmissible gastroenteritis virus, sequential vaccination

## Abstract

Porcine Epidemic Diarrhea Virus (PEDV) and Transmissible Gastroenteritis Virus (TGEV) pose significant threats to neonatal piglets, leading to severe diarrhea and potentially lethal consequences. Beyond enforcing stringent biosecurity protocols, effective and safe vaccinations are crucial in mitigating the impact of these diseases. In this study, the PEDV S1 (PS1) and TGEV S1 (TS1) antigens were initially chosen as candidates for the development of circRNA vaccines. Recognizing the comparatively lower immunogenicity of the PS1 antigen in contrast to the TS1 antigen, we strategically conjugated the PS1 with the pig fragment crystallizable (Fc) region to form PS1F. Despite these efforts, the bivalent circRNA vaccine prepared using an equal amount of the circRNA^PS1F^ and circRNA^TS1^ mixture still led to a reduction in the antibody levels against PS1. Subsequent dosage optimization of these two circRNA vaccines resulted in the induction of comparable levels of antigen specific antibodies and T cell immunity. Furthermore, sequential vaccination regimen with bivalent circRNA vaccine and commercial inactivated vaccines (IAV) could elicit a predominantly Th1-driven antibody responses and effectively neutralize both PEDV and TGEV. Our findings not only provide a potential strategy for the development of bivalent or multivalent circRNA/mRNA-based vaccines but also highlight the promising application of sequential vaccination strategies within the swine industry.

## 1 Introduction

PEDV and TGEV belong to the α-coronavirus genus and are classified as single-stranded positive-sense RNA viruses, both of which have been identified as the causative agents of pandemic swine diarrhea, leading to significant challenges in the porcine industry (1). The TGEV was initially documented in 1946 within the borders of the United States (2) and subsequently identified across Europe, Asia, Africa, and South America. The first detection of PEDV in Europe can be traced back to the early 1970s (3). Since 2010, highly pathogenic strains of PEDV have been gradually spreading within pig herds and persistently prevailing across various countries in Asia, North America, and Europe (4–8), particularly among neonatal piglets less than 10 days old who experience a mortality rate of 100% following PEDV infection (9). The widespread global distribution of both PEDV and TGEV has resulted in significant economic consequences for the global swine industry. The vaccination emerges as the most efficacious preventive measure in the face of intricate and dynamic diarrhea epidemics. Due to a severe co-infection of PEDV and TGEV, single vaccination cannot simultaneously provide protection against both virus infections (10). As a result, bivalent inactivated or attenuated live vaccines that target both PEDV and TGEV have been primarily employed for piglet protection. However, the effectiveness and safety of current vaccines are insufficient (11). Consequently, there is an urgent need to develop innovative bivalent vaccines that can offer both safety and potent efficacy against PEDV and TGEV.

The surface glycoprotein (S) of PEDV and TGEV is located in the outermost layer of the virion and shares functional similarities with the S protein of Severe Acute Respiratory Syndrome Coronavirus type 2 (SARS-CoV-2). The S protein plays a crucial role in binding to host cells, making its full-length or truncated forms significant targets for vaccine development (12, 13). Studies have shown that the core neutralizing epitope (COE) region of PEDV S1 has the function of producing neutralizing antibodies, while the N-terminal domain (NTD) region serves as potential co-receptor binding regions (12). The S1 domain of TGEV comprises four antigenic sites (or two antigenic sites), namely C, B, D, and A (or NTD and RBD) (14, 15), arranged sequentially from the N-terminus to the C-terminus. Among these sites, both A and D are capable of eliciting the production of neutralizing antibodies against TGEV in the host organism (14). Consequently, extensive utilization has been made of either the full-length S protein or specific domains such as S1 or COE region in immunogenic studies pertaining to PEDV or TGEV vaccines.

Recent studies have shown that mRNA vaccines have significant advantages over traditional vaccines (16), including flexibility in targeting emerging or mutated pathogens and the ability to quickly design, produce, and induce powerful immune responses (17, 18). During the COVID-19 pandemic, mRNA vaccines targeting SARS-COV-2 emerged as the pioneering choice for human immunization, significantly propelling the advancement of mRNA vaccine development, numerous mRNA vaccine candidates against other important pathogens have been under development (19–24), including vaccines against both human and animal pathogens. Since then, studies of PEDV mRNA vaccines have also been reported and produced promising immune effects (12). The primary limitation of mRNA vaccines, however, resides in their vulnerability to degradation by RNases, necessitating storage and transportation at low temperatures. This poses a significant challenge for veterinary vaccine development.

It has been established that circRNAs can be translated into proteins through a cap-independent mechanism (25), which enable their potential as a robust alternative to mRNA. Unlike linear mRNA, circRNA boasts exceptional stability due to its covalently closed circular configuration. Moreover, circRNA exhibits reduced immunogenicity and maintains the capacity for sustained and potent protein expression (26, 27), even in the absence of nucleotide modifications. Additionally, the absence of the need for capping and tailing reactions in circRNA synthesis further lowers production costs. These advantages make circRNA an attractive candidate for vaccine development, particularly in the veterinary field. Indeed, several studies have already been conducted on circRNA vaccines for coronavirus, rabies and mpox etc. (28–30), demonstrating that these vaccines can retain their integrity at room temperature for up to a month and effectively stimulate robust immune responses.

Given this, we developed circRNA vaccines targeting PEDV and TGEV, utilizing PS1 and TS1 as immunogens. Despite the inconsistent immunogenicity observed with individual circRNA^PS1^ and circRNA^TS1^ vaccines, we successfully obtain a bivalent vaccine with optimizing the antigen design and circRNA dosage ratio. This bivalent circRNA vaccine elicited comparable levels of B and T cell responses against both PEDV and TGEV. Our findings propose a promising dual-target strategy for combating both PEDV and TGEV viral infections simultaneously in the swine industry.

## 2 Materials and methods

### 2.1 Plasmid construction

The codon-optimized PS1, TS1, PS1F and TS1F genes were synthesized and cloned into the commercially available pCDNA3.4 plasmid by LOGENBIO Biological Company. Subsequently, the genes were integrated into a vector constructed for the synthesis of circular RNA, as per the methodology established in previous research by Wesselhoeft et al. A comprehensive description of all these sequences is provided in the **Supplementary Materials**.

### 2.2 Cells and mice

Expi293F cells (Thermo Fisher Scientific) were cultured in SMM 293-TII medium (Sino Biological) at 37°C under 5% CO_2_ in a shaking incubator and confirmed to be free of mycoplasma contamination. 6 to 8-week-old female BALB/c mice were purchased from Beijing Vital River Laboratory Animal Technology Co., LTD. The animals were housed under specific pathogen-free conditions in the Animal Care facility of Shanxi Agricultural University. All experimental procedures are conducted in strict accordance with the standards of the Institutional Animal Care and Use Committee of Shanxi Agricultural University (SXAU-EAW-2023M.DF.001017216).

### 2.3 CircRNA preparation and purification

The protocol for circRNA preparation follows the method as previous described (31). In brief, plasmid templates were linearized, and the resulting products were purified for subsequent IVT using the T7 High Yield RNA Synthesis Kit (Vazyme, China) at 37°C for 2 hours. Following this, the *in vitro* transcribed RNA products were treated with DNase I (Takara, Japan) to eliminate residual DNA, and Recombinant RNase Inhibitor (Takara, Japan) was introduced to mitigate RNA degradation. A subsequent thermal treatment at 70°C for 5 minutes was applied, after which GTP was incorporated to a final concentration of 2 mM, followed by the execution of an RNA circularization reaction at 55°C for 30 minutes. Finally, the RNA products were column-purified and analyzed on 2% agarose gels.

To purify the circRNA, a high-performance liquid chromatography (HPLC) assay was performed using a 4.6×300 mm size exclusion column with a particle size of 5 µm and pore size of 1000 Å (Sepax Technologies, USA) on an Elite EClassical 3200 Series HPLC system (Elite, China). RNA samples were run in RNase-free phosphate buffer (containing 150 mM sodium phosphate at pH 7.0) at a flow rate of 0.6 mL/minute. Detection and collection of RNA were carried out through UV absorbance measurement at a wavelength of 260 nm. Subsequently, the obtained RNA fractions underwent desalting using a dedicated column, followed by precipitation with ammonium acetate and resuspension in RNase-free water.

For splice junction analysis, the EasyScript One-Step gDNA Removal and cDNA Synthesis SuperMix (TransGen, China) was utilized for reverse transcription to synthesize cDNA using purified circRNA as a template. A standard PCR protocol with specific primer pairs spanning the splice junction of putative circRNA was utilized to amplify PCR products from synthetic cDNA templates. Then, these PCR products were cloned into pUC57 vectors for subsequent sequencing analysis.

### 2.4 DNA/RNA transfection

For expression analysis, 2 µg of plasmid DNA or 4 µg of HPLC-purified circRNA was transfected into 293F cells using polyethylenimine (PEI) as the transfection agent. The supernatants were harvested 48 hours post-transfection, and the expression of circRNA was evaluated by Western blotting.

### 2.5 Protein purification

Plasmids encoding the proteins of interest were mixed with PEI at a mass ratio of 1:4 and subsequently added to the culture medium containing 2 × 10^6^ Expi293F cells/mL. The supernatant was collected five days post-transfection and filtered through a 0.22 μm filter. PS1 and TS1 proteins were purified using a 5 mL HisTrap HP affinity column (GE Healthcare, USA), followed by further separation *via* gel filtration on a Superdex 200 10/300 GL column (GE Healthcare, USA). The molecular size and purity of the proteins were assessed by sodium dodecyl sulfate-polyacrylamide gel electrophoresis (SDS-PAGE).

### 2.6 CircRNA encapsulation

The circRNAs were encapsulated into lipid nanoparticles (LNPs) as previously delineated (31). Briefly, the aqueous phase containing circRNAs was mixed with an ethanolic solution of lipids and cholesterol in a microfluidic device at a ratio of 1:3 (v/v). Then, the circRNA@LNP complexes were subjected to dialysis in PBS for 12 hours (MWCO = 3.5 kDa). The size of LNP-circRNA complex was determined using dynamic light scattering (DLS) on Zetasizer Nano (Malvern Instruments, UK) and the morphology assays were carried out on a Hitachi HT-7700 transmission electron microscope (Hitachi, Japan).

### 2.7 Mouse immunization

The 6-8-week-old female BALB/c mice were immunized intramuscularly. The vaccination regimens and dosages of the commercial inactivated vaccine and circRNA-LNP vaccines were entailed in the accompanying figures. To evaluate the antibody responses, serum samples were collected biweekly following each vaccination. Additionally, spleens were harvested on day 7 post-booster immunization to evaluate T cell responses.

### 2.8 Enzyme-linked immunosorbent assay

ELISA plates were coated with either 2 μg/mL of PS1/TS1 recombinant protein or PEDV S1 specific antibody (LG3) in PBS buffer (pH 7.0), followed by blocking with 5% fat-free milk in PBS. Thereafter, diluted serum samples were introduced into the wells. Plates were incubated with goat anti-mouse IgG-HRP (Easybio, China), goat anti-mouse IgG1-HRP (Abcam, UK), goat anti-mouse IgG2a-HRP (Abcam, UK) or the PEDV S1 specific antibody conjugated with HRP (LB9-HRP), and developed with 3,3’,5,5’-tetramethylbenzidine (TMB) substrate. The reactions were stopped with 2 M H_2_SO_4_, and the absorbance at 450 nm was measured. The endpoint titer was defined as the highest reciprocal dilution of serum yielding an absorbance value exceeding 2.5-fold that of the background.

### 2.9 Neutralization assay

Vero-81 cells for PEDV or ST cells for TGEV were seeded into 96-well plates. Following this, the serum samples were inactivated at 56°C for 30 minutes and then serially diluted (in 2-fold increments) with cell culture medium. These diluted serum samples were subsequently mixed with 200 TCID_50_/50 μL of the corresponding virus and incubated at 37°C for 1 hour, prior to being added to the 96-well plate containing the respective cells. The cells were monitored every 24 hours, and the observations of cytopathic effects (CPE) were recorded. The neutralizing antibody titer was calculated based on the Reed-Muench method as previously described (32).

### 2.10 Flow cytometry analysis

Spleens harvested from immunized mice were processed to generate single-cell suspensions, from which 2 x10^6^ cells per sample were seeded into 96-well plates. 10 μg/mL of either PS1 or TS1 protein was utilized to stimulate the splenocytes. Then Brefeldin A Solution (BFA) was added to each well and incubated at 37°C for 6 hours to inhibit protein secretion and facilitate the detection of intracellular cytokines. Anti-FcgIII/II receptor (clone 2.4G2) was used to prevent non-specific binding. Fixable Viability Dye eFluor™ 450 (eBioscience, USA) was applied to exclude dead cells from the analysis. For surface staining, APC anti-mouse CD8α (BioLegend, USA) was used to identify CD8^+^ T cells, while intracellular staining for PE anti-mouse IFN-γ (BioLegend, USA) was performed using the True-Nuclear™ transcription factor buffer set (BioLegend, USA). Data acquisition was performed on a CytoFLEX flow cytometer (Beckman Coulter, USA), and with subsequent data analysis using CytExpert software (BeckmanCoulter, USA).

### 2.11 *In vivo* toxicity analysis

6-8-week-old female BALB/c mice were immunized with LNP or the bivalent circRNA vaccine comprising 20 μg of circRNA^PS1F^ and 5 μg of circRNA^TS1^. Post-immunization, the body weight changes were monitored over a 14-day period. Serum samples were collected 48 hours after immunization to assess the levels of alanine aminotransferase (ALT), aspartate aminotransferase (AST), and creatinine (CREA) were determined. 7 days after the booster immunization, tissue samples from the heart, liver, spleen, lung, and kidney were harvested to hematoxylin and eosin staining for histopathological examination.

### 2.12 Statistical analysis

The data in this study were presented as mean ± S.D. Unpaired two-tailed Student’s *t*-tests were utilized for comparative analyses and *P* < 0.05 was considered statistically significant.

## 3 Results

### 3.1 Production and characterization of circRNA^PS1^ and circRNA^TS1^ vaccines

**Figure 1:**
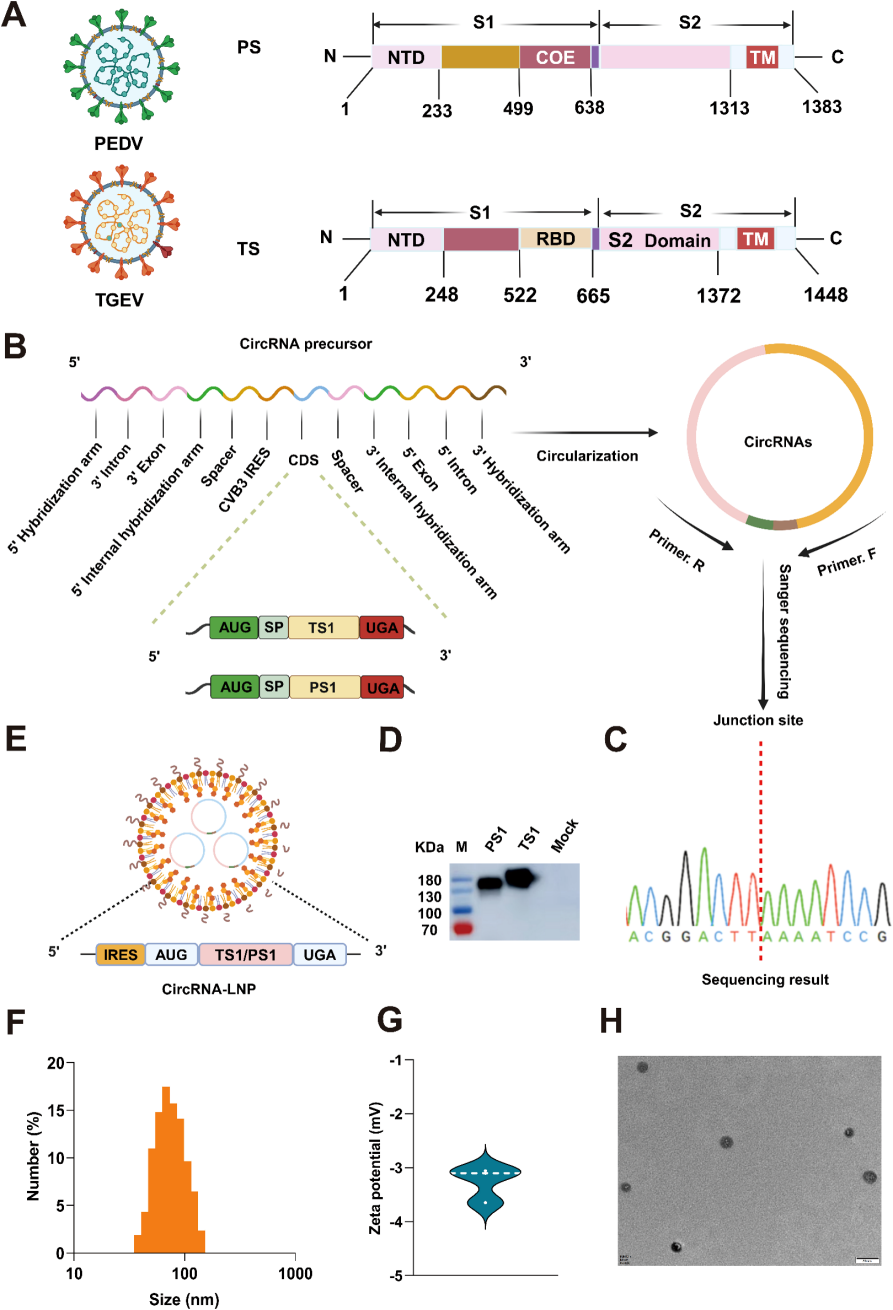
Production and characterization of circRNA^PS1^ and circRNA^TS1^ vaccines. **(A)** Schematic representation of the spike (S) protein from PEDV or TGEV. PS: S protein of PEDV; TS: S protein of TGEV. **(B)** The genes encoding the PS1 and TS1 domains were optimized, synthesized and inserted into the vector with PIE system for circRNA production *in vitro*. SP: Signal peptide. **(C)** The RT-PCR products of circRNA^TS1^ and circRNA^PS1^ were analyzed by Sanger sequencing to validate the splicing sites of the circRNAs. **(D)** The expression levels of PS1 and TS1 in the supernatants were determined by western blot analysis at 48 hours post-transfection of 293F cells with circRNA^TS1^ and circRNA^PS1^. **(E)** Schematic depiction of the circRNA-LNP complex. **(F)** Size distributions of the circRNA-LNP complex. **(G)** Zeta potential of the circRNA-LNP complex. **(H)** TEM image of the circRNA-LNP complex. Scale bar, 50 nm.

Given that the S1 region of the S protein is primarily responsible for generating neutralizing antibodies against PEDV and TGEV (**Figure 1A**), the respective coding sequence of S1 was optimized and inserted into the well-known permuting intron-exon (PIE) system derived from Anabaena pre-tRNA^Leu^ gene developed by Wesselhoeft et al. (27) to synthesize circRNA *in vitro* (**Figure 1B**). To ensure high purity of the circRNA, a purification procedure utilizing high-performance liquid chromatography (HPLC) was employed (**Supplementary Figures S1A, B**). After conducting a meticulous circRNA collection, we performed desalination and precipitation procedures to obtain high-quality circRNA^PS1^ and circRNA^TS1^. Their quality was further confirmed through agarose gel electrophoresis and HPLC analysis (**Supplementary Figures S1C, D**). In addition, we calculated that the circularization efficiency of both types of circular RNAs exceeded 80% (**Supplementary Figure S1E**).

Next, the circRNA samples were subjected to reverse transcription, followed by PCR amplification using primer pairs spanning the junction site and subsequent Sanger sequencing, to validate the accuracy of the splicing reaction. The results confirmed that both circRNA^PS1^ and circRNA^TS1^ underwent precise splicing as anticipated (**Figure 1C**). Subsequently, the circRNA^PS1^ and circRNA^TS1^ were transfected into 293F cells to assess their expression capacity *in vitro*, and the results demonstrated successful *in vitro* expression of both circRNAs (**Figure 1D**).

Then, the circRNA^PS1^ and circRNA^TS1^ were encapsulated in lipid nanoparticles (LNPs) to form circRNA-LNP complexes (**Figure 1E**). Dynamic light scattering (DLS) measurements revealed an average particle size of approximately 72.5 nm (**Figure 1F**), with a corresponding average zeta potential of approximately -3.3 mV (**Figure 1G**). Transmission electron microscopy (TEM) evaluation confirmed the spherical nanoparticle morphology of the circRNA-LNP complex (**Figure 1H**).

### 3.2 The circRNA^PS1^ and circRNA^TS1^ vaccines stimulate antibody production in a dose-dependent manner

**Figure 2:**
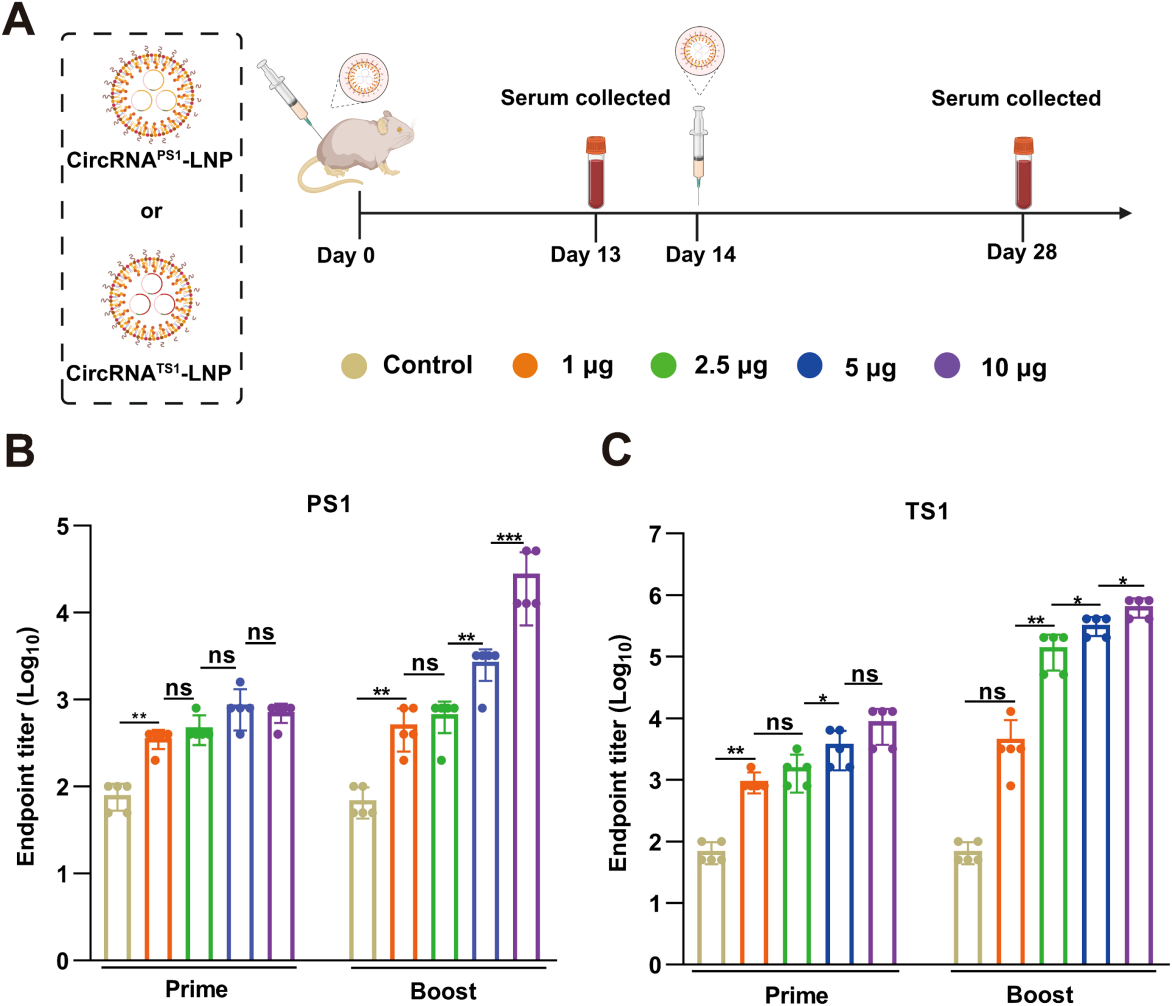
Immunogenicity assessment of the circRNA^PS1^ and circRNA^TS1^ vaccines. **(A)** Schematic illustration of the immunogenicity assessment protocol for circRNA^PS1^ and circRNA^TS1^ vaccinations. **(B, C)** Quantitative analysis of PS1-specific **(B)** and TS1-specific **(C)** IgG antibody titers induced by the circRNA^PS1^ and circRNA^TS1^ vaccines, respectively, at the indicated dosages. These values are expressed as mean ± S.D, n=5. (ns, *P < 0.05, **P < 0.01, ***P < 0.001).

To evaluate the immunogenicity of the two circRNA vaccines, we initially immunized BALB/c female mice *via* intramuscular injection with different doses of circRNA^PS1^-LNP and circRNA^TS1^-LNP. The vaccination protocol involved two doses administered with a two-week gap between the initial and the booster immunization (**Figure 2A**). Serum samples were collected 13 days post each immunization to perform enzyme-linked immunosorbent assay (ELISA) analyses for the quantification of specific antibodies against PS1 and TS1 antigens (**Figures 2B, C**). The results revealed that the serum antibody levels against both TS1 and PS1 escalated in a dose-dependent fashion. Notably, when the circRNA^TS1^ dose surpassed 2.5 μg, there was a steady rise in TS1-specific antibodies, culminating in endpoint titers nearing 10^6^ in the cohort that received the highest vaccine dosage of 10 μg (**Figure 2C**). In contrast, for circRNA^PS1^, a dose of over 5 μg triggered a marked increase in PS1-specific antibodies in the serum (**Figure 2B**). However, a comparison between the groups that received the maximum dose of 10 μg revealed that the average PS1-specific antibody level was approximately one-tenth of that for TS1 (**Figures 2B, C**). These findings suggest that the immunogenicity of PS1 is comparatively lower, and improving the immunogenicity of PS1 could significantly advance the development of a bivalent vaccine against PEDV and TGEV.

### 3.3 The Fc fusion significantly amplifies the immunogenicity of PS1 in the form of circRNA

**Figure 3:**
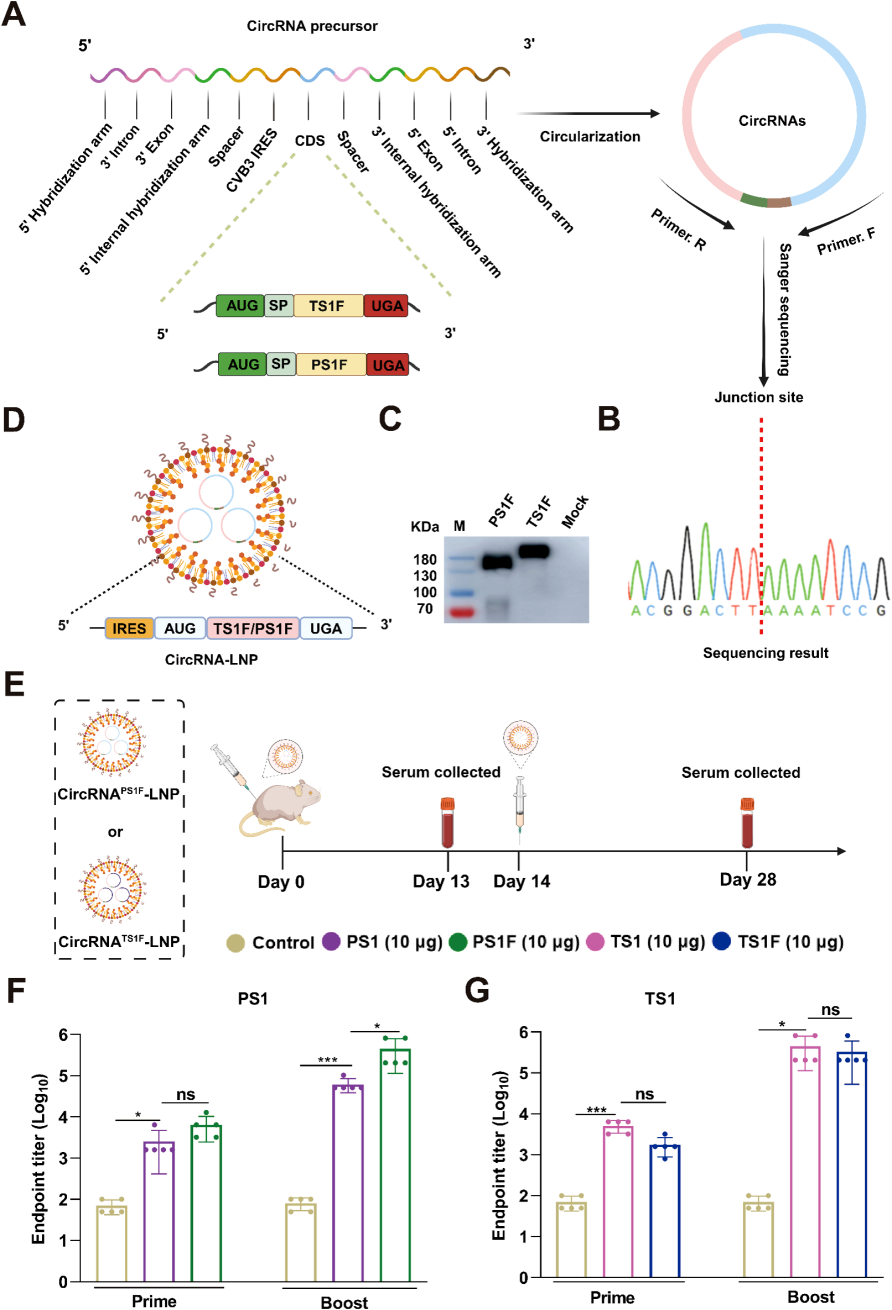
The Fc fusion significantly amplifies the immunogenicity of PS1 in the form of circRNA. **(A)** Schematic illustration of the preparation of circRNA**^PS1F^** and circRNA**^TS1F^**. **(B)** The RT-PCR products of circRNA^PS1F^ and circRNA^TS1F^ were analyzed by Sanger sequencing to validate the splicing sites of the circRNAs. **(C)** The expression levels of PS1F and TS1F in the supernatants were determined by western blot analysis at 48 hours post-transfection of 293F cells with circRNA^PS1F^ and circRNA^TS1F^. **(D)** Schematic depiction of the circRNA^PS1F/TS1F^-LNP complex. **(E)** Schematic representation of the vaccination regimen with circRNA^PS1F^ and circRNA^TS1F^. An equivalent dosage of circRNA^PS1/TS1^-LNP or LNP control was administered following the same protocol. **(F, G)** Quantitative analysis of PS1-specific **(F)** and TS1-specific **(G)** IgG antibody titers post-vaccination. These values are expressed as mean ± S.D, n=5. (ns, *P < 0.05, ***P < 0.001).

Previous studies have revealed that incorporating the Fc region into an antigen can significantly boost the immunogenicity of vaccines (33, 34). Building on this, we conducted a redesign of circRNA sequences encoding fusion proteins, attaching a porcine Fc region at the C-terminus of both PS1 and TS1 (**Figure 3A**). Following the preparation process, we successfully obtained two circRNAs, designated as circRNA^PS1F^ and circRNA^TS1F^ (**Figure 3B**, **Supplementary Figures S2A-S2D**). In addition, we calculated that the circularization efficiency of both types of circular RNAs exceeded 80% (**Supplementary Figure S2E**). The expression of these circRNAs was then validated by western blot (**Figure 3C**). After encapsulation in LNP (**Figure 3D**), we intramuscularly injected two circRNA vaccines into female BALB/c mice, each containing 10 μg of circRNA^PS1F^ and 10 μg of circRNA^TS1F^, with equivalent doses of circRNA^PS1^ and circRNA^TS1^ used as controls (**Figure 3E**). The analysis of serum samples, collected after both the primary and booster immunizations, demonstrated a marked increase in the immunogenicity of circRNA^PS1F^ vaccine compared to the circRNA^PS1^ vaccine, with endpoint titers against PS1 reaching approximately 10^6^ (**Figure 3F**). In addition, on day 100 post-booster immunization, serum samples were collected and analyzed for anti-PS1 antibody levels using ELISA. The results showed that the antibody titers induced by the circular RNA vaccine were maintained at a relatively high level. (**Supplementary Figure S3**). Unexpectedly, however, the fusion of an Fc tag with TS1 antigen in the circRNA vaccine did not augment its immunogenicity (**Figure 3G**). All these results indicated that circRNA^PS1F^ and circRNA^TS1^ should be used for subsequent bivalent vaccine development.

### 3.4 Equal dosing of the circRNA^PS1F^ and circRNA^TS1^ vaccines results in a diminished antibody response against PS1

**Figure 4:**
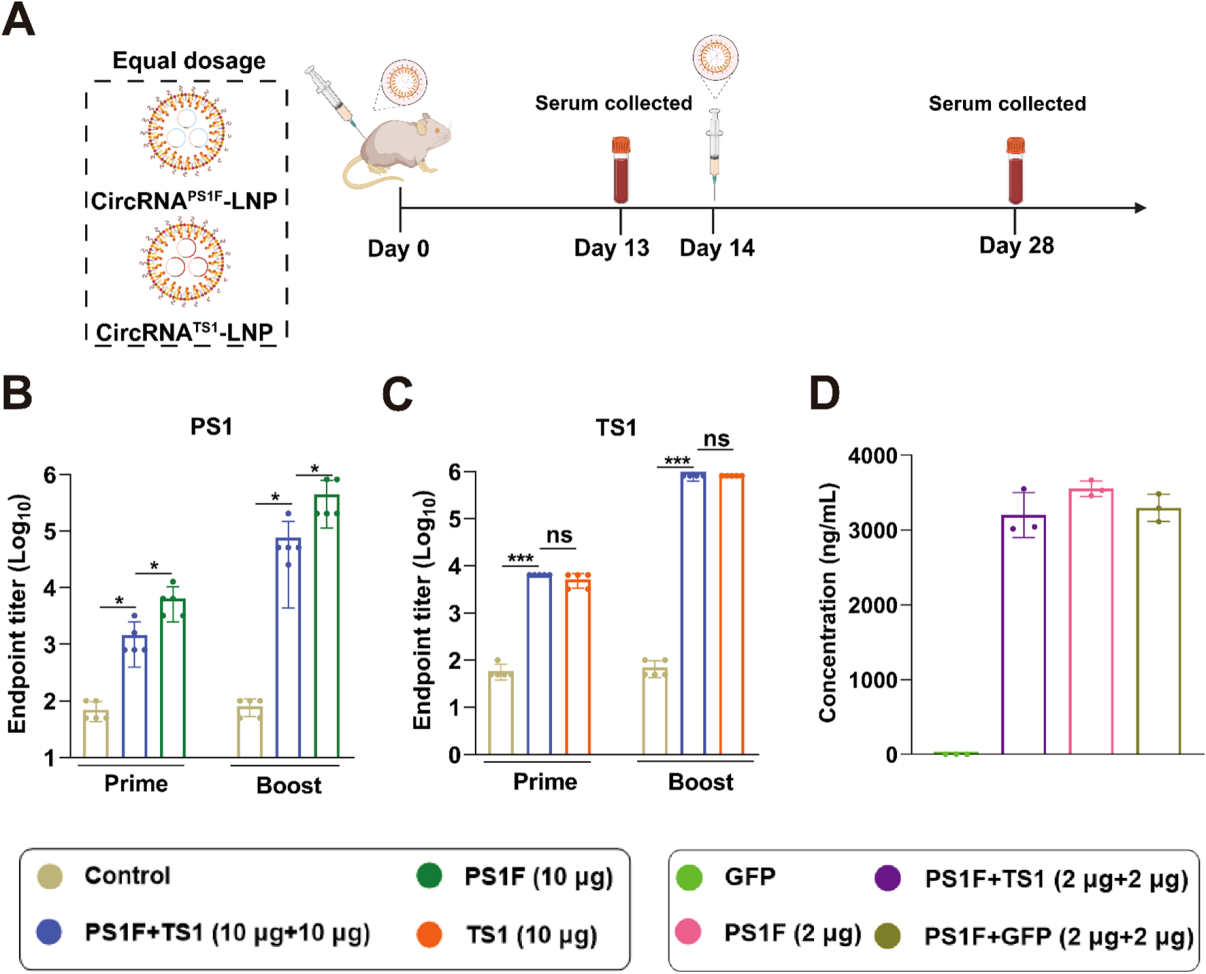
Equal dosing of the circRNA^PS1F^ and circRNA^TS1^ vaccines leads to i a significant reduction in antibody levels against PS1. **(A)** Schematic depiction of the vaccination regimen. A mixture of circRNA^PS1F^ (10 μg) and circRNA^TS1^ (10 μg) was administered in accordance with the outlined protocol. Additionally, CircRNA^PS1F^ (10 μg), CircRNA^TS1^ (10 μg), or LNP (as a control) was delivered following the identical vaccination regimen. **(B-C)** Quantitative analysis of PS1-specific **(B)** and TS1-specific **(C)** IgG antibody titers. **(D)** Expression analysis of PSIF was performed 48 hours after transfection of 293F cells with the specified circRNAs. These values are expressed as mean ± S.D, n=5. (ns, *P < 0.05, ***P < 0.001).

In the pursuit of developing a bivalent vaccine, we initially co-administered equal dosing (10 μg circRNA) of circRNA^PS1F^-LNP and circRNA^TS1^-LNP, employing an immunization protocol as described above. Concurrently, individual administrations of circRNA^PS1F^-LNP (10 μg) and circRNA^TS1^-LNP (10 μg) served as respective controls (**Figure 4A**). Subsequent quantitative analyses of serum samples using ELISA revealed that, following booster immunization, the bivalent vaccine group demonstrated a significantly reduced presence of specific antibodies targeting PS1 when compared to the standalone circRNA^PS1F^-LNP vaccine (**Figure 4B**). Conversely, no significant disparity was observed in the levels of specific antibodies against TS1 between the bivalent vaccine group and the circRNA^TS1^-LNP vaccine group (**Figure 4C**). These findings indicate that while the immunogenicity of PS1 antigen was augmented by incorporating Fc fragment, achieving a comparable level to that of the TS1 antigen, co-administration of circRNA^PS1F^-LNP and circRNA^TS1^-LNP still led to a pronounced decrease in the induction of antibodies specific to PS1.

To ascertain the effect of co-transfection of circRNA^PS1F^ and circRNA^TS1^ on PS1F antigen expression, 293F cells were co-transfected with equal amounts of circRNA^PS1F^ and circRNA^TS1^, after which the expression of the PS1F antigen was evaluated. The results demonstrated that the presence of circRNA^TS1^ did not exert a significantly influence on the expression of the PS1F derived from circRNA^PS1F^, implying that the observed disparities in the antibody response towards PS1F and TS1 *in vivo* could be ascribed to antigenic competition (**Figure 4D**). Therefore, this bivalent vaccine formulation should be further optimized to achieve robust antibody responses against both antigens.

### 3.5 Optimize the dose ratio of bivalent vaccines to induce robust humoral and cellular immune responses against both PEDV and TGEV

**Figure 5:**
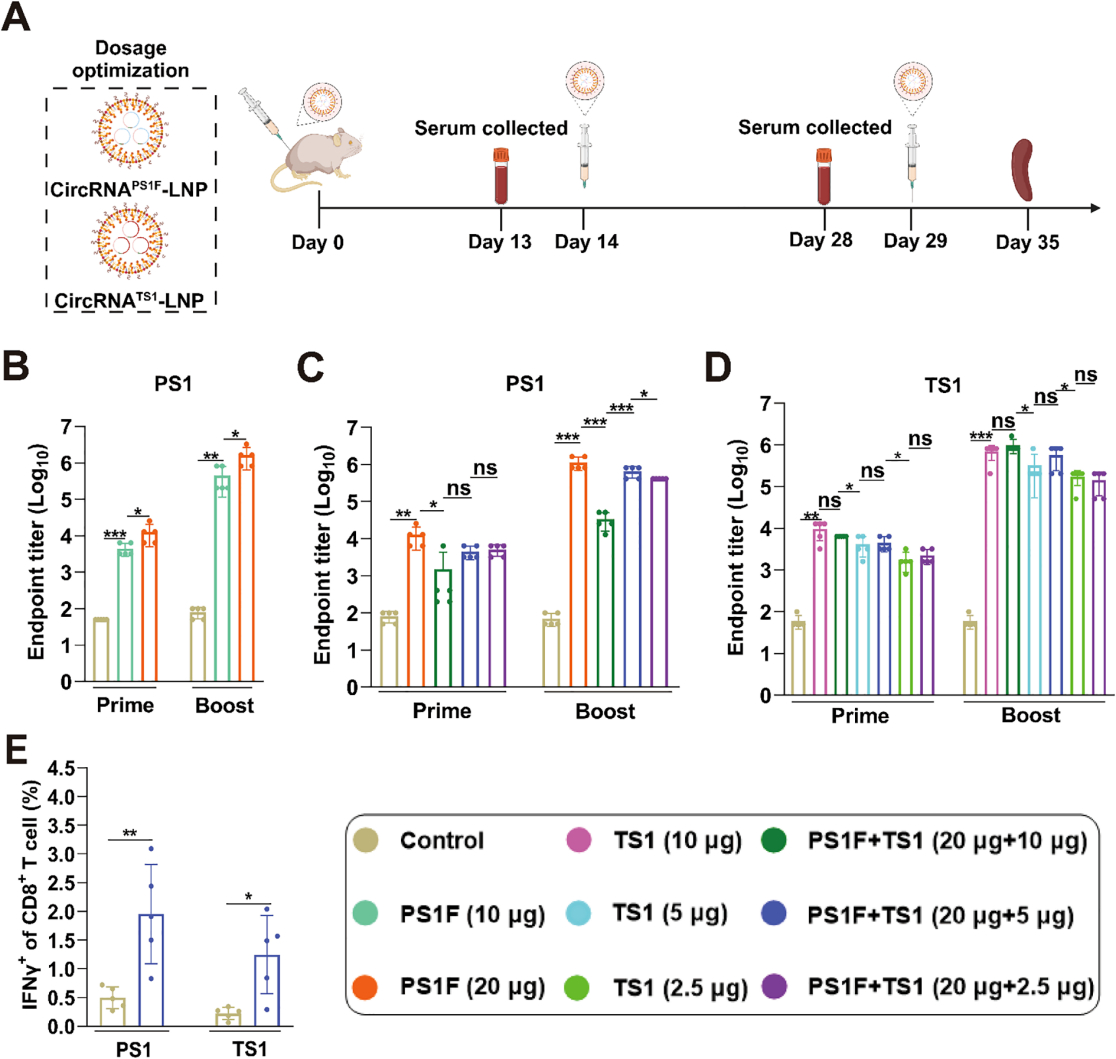
Optimize the dose ratio of bivalent vaccines to induce higher levels of humoral and cellular immune responses against both PEDV and TGEV. **(A)** Schematic depiction of the immunization regimen for the bivalent circRNA vaccine. **(B)** Quantitative analysis of PS1-specific IgG antibody titers induced by circRNA^PS1F^ vaccine (10 μg or 20 μg). **(C, D)** Assessment of PS1 or TS1-specific IgG antibody titers induced by bivalent vaccines comprising circRNA^PS1F^-LNP (20 μg) and circRNA^TS1^-LNP (0, 2.5, 5 or 10 μg). **(E)** Detection of PS1 and TS1 antigen-specific T cell responses, as indicated by IFN-γ^+^ CD8^+^ T cells, in the splenocytes from mice immunized with bivalent vaccine comprising circRNA^PS1F^ (20 μg) and circRNA^TS1^ (5 μg). These values are expressed as mean ± S.D, n=5. (ns, *P < 0.05, **P < 0.01, ***P < 0.001).

To ameliorate the variances in the antibody response elicited by the bivalent vaccine, we undertook the optimization of circRNA dosages. The antibody induction capacity of 20 μg circRNA^PS1F^ was initially evaluated (**Figure 5A, B**), with outcomes revealing a markedly elevated induction of PS1 specific antibodies by 20 μg circRNA^PS1F^ compared to that induced by the 10 μg circRNA^PS1F^. Subsequently, we administered circRNA^PS1F^-LNP (20 μg) with varying dosages of circRNA^TS1^-LNP (2.5, 5, or 10 μg) in mice. The ensuing data indicated that co-administration of circRNA^PS1F^-LNP (20 μg) with 10 μg of circRNA^TS1^ significantly influenced the production of PS1 antibody; however, the co-administration of circRNA^PS1F^-LNP (20 μg) with 2.5 μg and 5 μg of circRNA^TS1^ did not result in significant suppression (**Figure 5C**). Furthermore, the bivalent vaccines containing 2.5, 5, or 10 μg of circRNA^TS1^ elicited comparable levels of TS1 antibody to the respective standalone circRNA^TS1^-LNP vaccines (**Figure 5D**). Informed by these observations, we formulated an optimized bivalent vaccine candidate, comprising circRNA^PS1F^-LNP (20 μg) and circRNA^TS1^-LNP (5 μg), simultaneously targeting both PEDV and TGEV.

Following the booster immunization with the optimal bivalent vaccine, we assessed the CD8^+^ T cell immune response induced by this vaccine combination. Flow cytometry analysis revealed that significant T cell responses that were specifically generated against PEDV and TGEV upon antigenic stimulation (**Figure 5E**, **Supplementary Figure S4**). Collectively, these findings indicate that the optimized bivalent vaccine presents a promising dual-target strategy for the simultaneous mitigation of both PEDV and TGEV infections within the swine industry.

### 3.6 Safety assessment of the bivalent vaccine candidate

The safety profile of the bivalent vaccine candidate was evaluated in immunized mice. Monitoring of post-initial immunization body weight changes over a 14-day period demonstrated that, after an initial phase of weight reduction, the immunized mice exhibited a comparable weight change to that of the control group (**Supplementary Figure S5A**). Serum samples obtained 48 hours post-booster immunization were analyzed for hepatic, and renal function, with results showing no significant deviations from the normal range (**Supplementary Figures S5B-S5D**). Furthermore, histopathological assessments of the heart, liver, spleen, lung, and kidney tissues from the immunized mice showed no notable pathological changes after administration of the bivalent circRNA vaccine (**Supplementary Figure S5E**). These observations collectively substantiate the safety profile of the circRNA vaccines, supporting their potential for clinical use.

### 3.7 Sequential vaccination elicits robust immunity against both PEDV and TGEV

**Figure 6:**
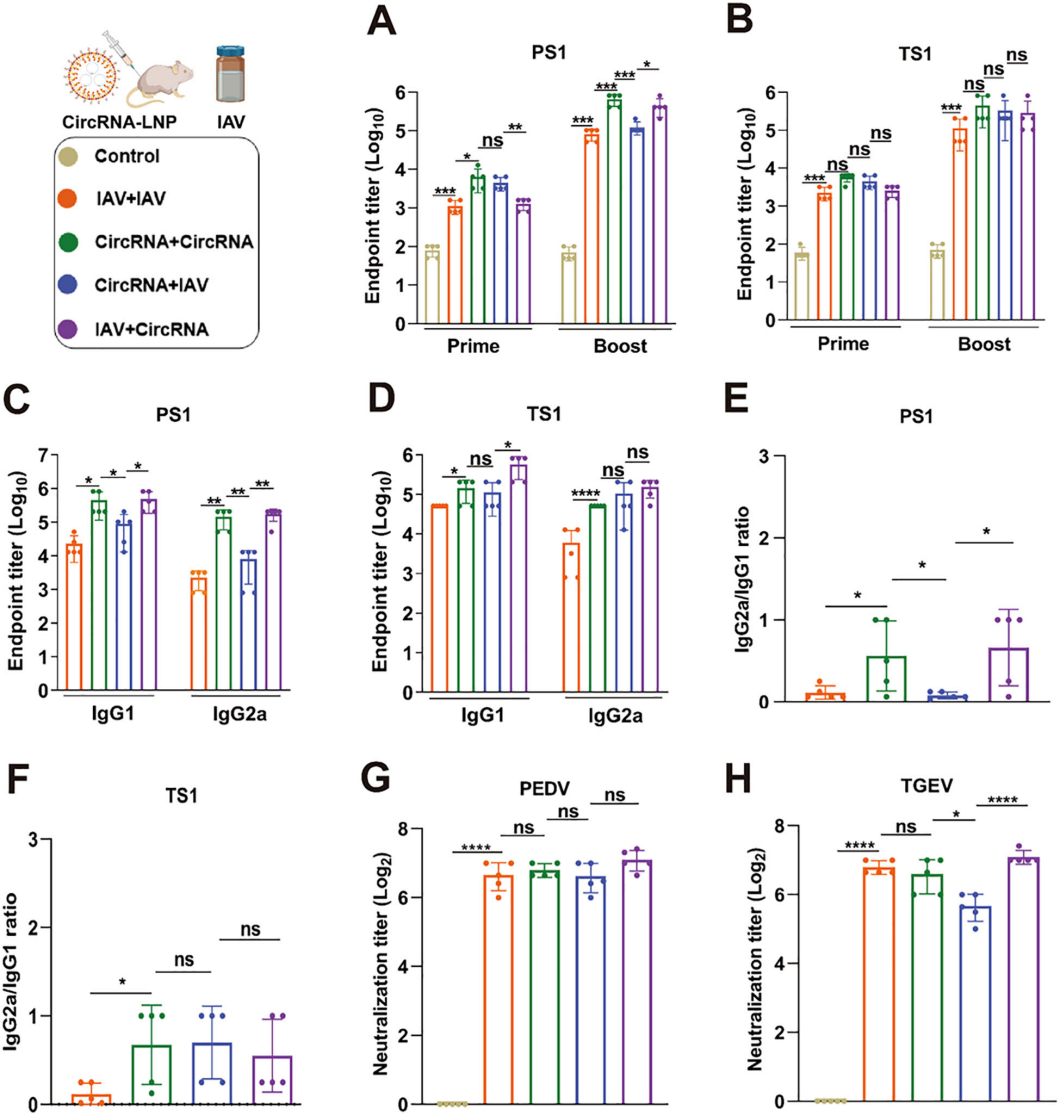
Sequential vaccination elicits robust immunity against both PEDV and TGEV. **(A, B)** Quantitative analysis of PS1-specific **(A)** and TS1-specific **(B)** IgG antibody titers in serum samples from mice with various immunization strategies. **(C, D)** Assessment of PS1-specific **(C)** and TS1-specific **(D)** IgG1 and IgG2a antibody titers. **(E, F)** Calculation of the IgG2a/IgG1 ratios for PS1-specific **(E)** and TS1-specific **(F)** antibodies, as presented in panels **(C)** and **(D)**. **(G, H)** Neutralization assays of authentic PEDV and TGEV were carried out using the serum from mice immunized with corresponding strategies. IAV: Commercially available inactivated bivalent vaccine against PEDV and TGEV; CircRNA: The optimized bivalent circRNA vaccine comprising 20 μg of circRNA^PS1F^ and 5 μg of circRNA^TS1^. These values are expressed as mean ± S.D, n=5. (ns, *P < 0.05, **P < 0.01, ***P < 0.001, ****P < 0.0001).

Previous studies have demonstrated that sequential vaccination strategies elicit a heightened immune response against infectious agents as opposed to homologous vaccination regimens (35). Building upon these findings, we have elected to explore the potential of our circRNA-based bivalent vaccine when administered in tandem with commercially available inactivated vaccines (IAV) to ascertain whether this combinatorial approach can evoke a more potent immune response and confer superior protection against PEDV and TGEV. Within the context of prime-boost immunization, four discrete vaccination regimens were designed: IAV-IAV, circRNA-circRNA, IAV-circRNA, and circRNA-IAV. Following the immunization protocols as delineated in **Figure 5A**, specific antibodies elicited against PS1 and TS1 in serum samples were detected. The results revealed that both the IAV-circRNA and circRNA-circRNA vaccine groups exhibited markedly elevated antibody titers against PS1 compared to the other two groups (**Figure 6A**). Conversely, the antibody responses against TS1 were found to be similar among all four vaccine groups (**Figure 6B**).

Additionally, we conducted a detailed analysis to investigate the specific IgG1 and IgG2a antibodies induced by these four immunization strategies. In the assessment of specific IgG1 and IgG2a antibody levels against PS1 (**Figure 6C**), both the IAV-IAV and circRNA-IAV groups exhibited a Th2-biased antibody response. In contrast, the IAV-circRNA and circRNA-circRNA groups exhibited a tendency towards a Th1-biased response (**Figure 6E**). Upon evaluating the specific IgG1 and IgG2a antibody levels against TS1 (**Figure 6D**), only the IAV-IAV immunization group manifested a Th2-biased humoral response, whereas the remaining three groups demonstrated a more pronounced inclination towards a Th1-biased response (**Figure 6F**). These observations imply that circRNA vaccine formulation possesses a significant advantage in eliciting a Th1-biased immune response.

Furthermore, we conducted neutralization assays against authentic PEDV and TGEV, and the results revealed that the IAV-circRNA group exhibited a significantly elevated neutralization titer against TGEV when compared to the other three groups. Conversely, the neutralization effects against PEDV were found to be analogous across all four groups (**Figures 6G, H**). Collectively, the sequential vaccination procedure employed in the IAV-circRNA group emerges as an optimal approach for the protection against PEDV and TGEV infections within the porcine industry.

## 4 Discussion

The predominant clinical signs of PEDV and TGEV infections include vomiting and diarrhea, which lead to escalated mortality rates among piglets during production practices (9). In recent years, concurrent infections of PEDV and TGEV have been frequently observed clinically (36–39). The coexistence of these diseases not only exerts immense pressure on farms for prevention and control but also incurs substantial economic losses. Consequently, the development of bivalent vaccines that target both viruses has become an urgent necessity. Our current study demonstrates the development of a bivalent circRNA vaccine against both PEDV and TGEV through rational antigen design and vaccination dose optimization. This bivalent vaccine elicits robust humoral and cellular immune responses, and serum from immunized mice can effectively neutralize both PEDV and TGEV, underscoring its potential application in the swine industry to provide comprehensive protection against these infections.

Initially, TS1 and PS1 domains were selected as immunogens for circRNA vaccine design. The subsequent results indicated that both vaccines exhibited robust immunogenicity, particularly circRNA^TS1^, which effectively induced high levels of antibody titers in mice even at lower doses. In contrast, circRNA^PS1^ produced comparatively weaker antibody titers. To bolster the immunogenicity of the circRNA^PS1^ vaccine, we refined the PS1 antigen by incorporating a pFc region (33, 34). This strategic modification enhanced its immunogenicity to a level comparable with that of circRNA^TS1^, thereby establishing a crucial foundation for developing an efficient bivalent circRNA vaccine capable of combating both PEDV and TGEV.

However, when equal doses of circRNA^TS1^ and circRNA^PS1F^ were combined into a bivalent vaccine and administered, a notable difference was observed in their capacity to elicit antibody responses. The phenomenon described above has previously been observed in the development of bivalent mRNA vaccines for RSV and SARS-CoV2 (40). Additionally, recent studies on multi-valent mRNA vaccines for mpox have shown that the simultaneous administration of different antigens in equal doses can lead to considerable variations in the antibody titers against each antigen (41). Such variability could significantly diminish the efficacy of the multi-valent vaccine, potentially negating the advantages of employing bivalent or multi-valent vaccine formulations. In the context of this study, the capacity of circRNA^PS1F^ to induce antibody response was considerably compromised when it was administered as part of the bivalent circRNA vaccine. It is evident from our data that this suppression is not due to antigen expression levels, implying that our bivalent circRNA vaccine indeed confronts challenges related to antigenic competition. However, the underlying mechanisms driving this phenomenon are not yet fully understood. Although, we have successfully addressed this issue by conjugating the PS1 with the pFc region and optimizing the dosage ratio of circRNA^TS1^ and circRNA^PS1F^, it serves as a cautionary note that antigenic competition (40) must not be disregarded, and greater efforts should be dedicated to elicit sufficient antibody responses against all the immunogens during the development of bivalent and multivalent vaccines.

During the 2019 COVID-19 pandemic, numerous studies have demonstrated that sequential vaccination using SARS-CoV-2 vaccines developed through various technologies yielded superior protective effects against infections (35). Dong et al. also found that a sequential vaccination strategy offered optimal cross-protection against influenza strains that have undergone antigenic drift and metastasis (42). In our current research, we have explored various immunization strategies using a bivalent circRNA vaccine in combination with commercially available inactivated vaccines (IAV). The findings revealed that both the circRNA-circRNA and IAV-circRNA vaccination groups produced higher total IgG, IgG1, and IgG2a antibody levels against the PS1 antigen compared to the IAV-IAV and circRNA-IAV groups, while all four vaccination strategies induced similar antibody levels against TS1, which may be due to its superior immunogenicity. Furthermore, immunization regimens incorporating circRNA vaccines were found to elicit Th1-biased immune responses. Notably, the IAV-circRNA group showed a higher total IgG level and a more pronounced Th1-biased antibody response against PS1 than the circRNA-IAV group. Additionally, the IAV-circRNA group demonstrated more effective neutralization of TGEV compared to the circRNA-IAV group. These observations underscore the potential of sequential vaccination strategies to offer enhanced protection against viral infections and highlight the importance of selecting the appropriate sequence of different vaccines for optimal efficacy.

In summary, our study has successfully developed a potent bivalent circRNA vaccine and a sequential strategy that capable of offering protection against both individual or co-infections of PEDV and TGEV. Beyond PEDV and TGEV co-infections, extensive epidemiological investigations on porcine diarrheal virus disease have revealed that the predominant forms of co-infection also included PEDV-Porcine delta corona virus (PDCoV), PEDV-porcine rotavirus (PoRV), and PEDV-TGEV-PoRV (1, 36–39). This complex landscape poses a significant challenge for the prevention and control of porcine diarrheal virus disease. Our research on the bivalent circular RNA vaccine platform, and the sequential immunization approach provides crucial insights and practical recommendations for the future development of bivalent and multivalent vaccines to combat these potential co-infections of porcine diarrheal viruses and greatly benefit the swine industry.

## Data Availability

The datasets presented in this study can be found in online repositories. The names of the repository/repositories and accession number(s) can be found in the article/**Supplementary Material**.
