# Supplementary material for: Bivalent circular RNA vaccines against porcine epidemic diarrhea virus and transmissible gastroenteritis virus
Source: Front Immunol. 2025 Mar 31;16:1562865. doi: 10.3389/fimmu.2025.1562865 (PMC11994721; doi:10.3389/fimmu.2025.1562865)
Supplement: Supplementary file 2 [file DataSheet1.docx]

Supplementary Material


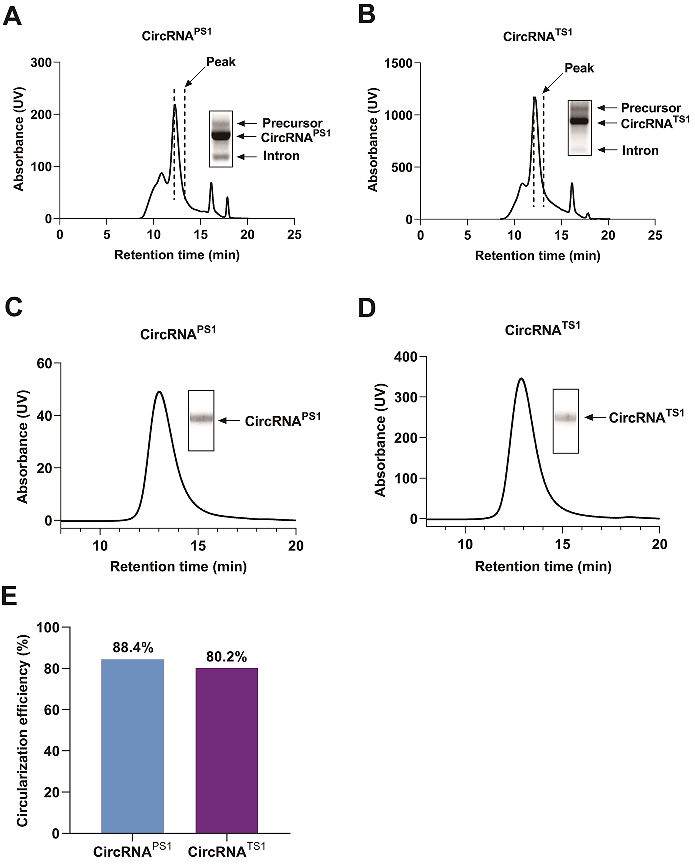


**Figure S1. Purification of circRNA^PS1^ and circRNA^TS1^.** (A-B) HPLC chromatogram and agarose gel electrophoresis analysis of the synthesized circRNA^PS1^ (A) and circRNA^TS1^ (B), respectively. The retention time corresponding to the collection of samples has been indicated. (C-D) HPLC and agarose gel electrophoresis analysis of purified circRNA^PS1^ (C) and circRNA^TS1^ (D). (E) Circularization efficiency of circRNA^PS1^ and circRNA^TS1^ was analyzed using Image J software.


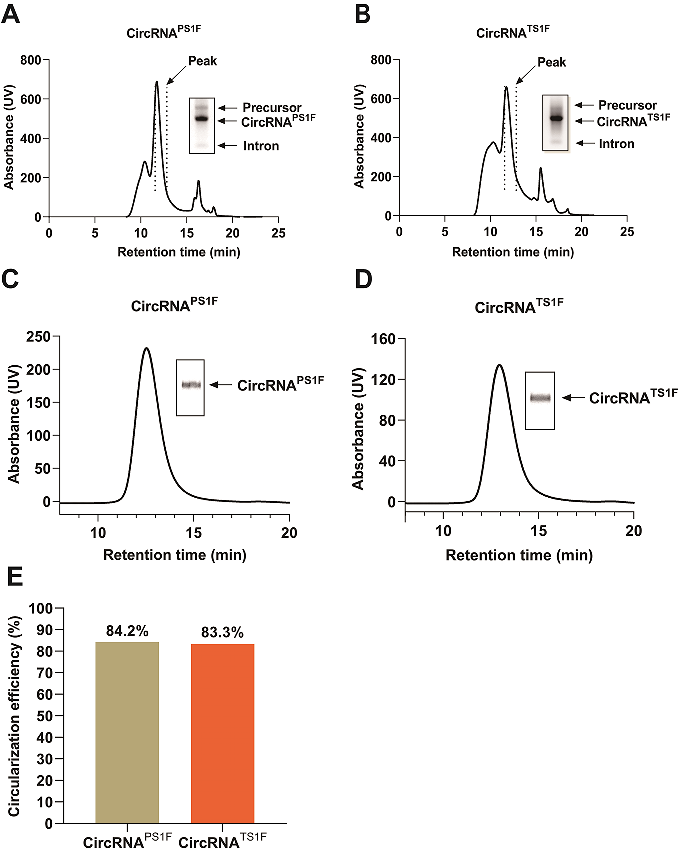


**Figure S2. Purification of circRNA^PS1F^ and circRNA^TS1F^.** (A-B) HPLC chromatogram and agarose gel electrophoresis analysis of the synthesized circRNA^PS1F^ (A) and circRNA^TS1F^ (B), respectively. The retention time corresponding to the collection of samples has been indicated. (C-D) HPLC and agarose gel electrophoresis analysis of purified circRNA^PS1F^ (C) and circRNA^TS1F^ (D). (E) Circularization efficiency of circRNA^PS1F^ and circRNA^TS1F^ was analyzed using Image J software.


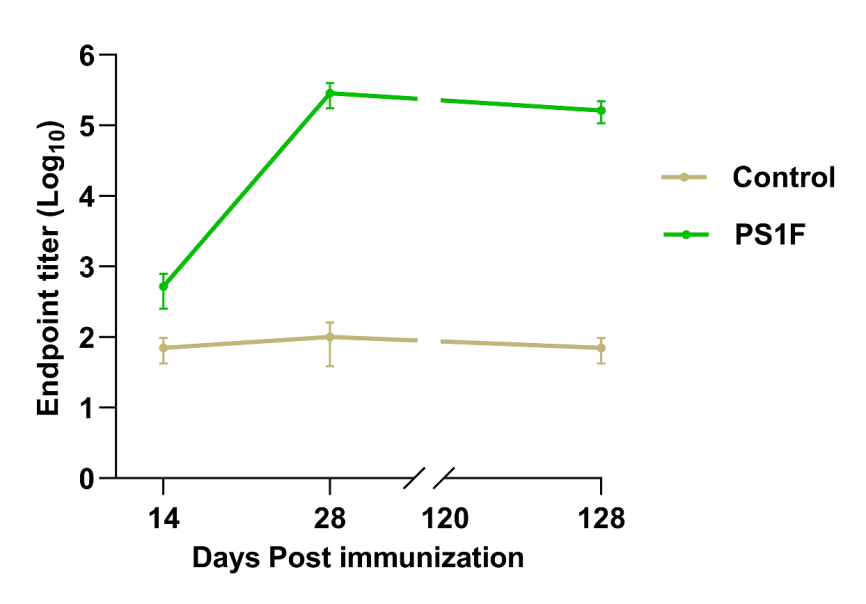


**Figure S3. CircRNA vaccine elicited a long-lasting and specific antibody response.** ELISA analysis was conducted to evaluate the specific antibody titers against PS1 in the serum of mice immunized with the circRNA^PS1F^ vaccine on days 14 and 100 post-boost immunization. The results are presented as mean ± S.D, n=5.


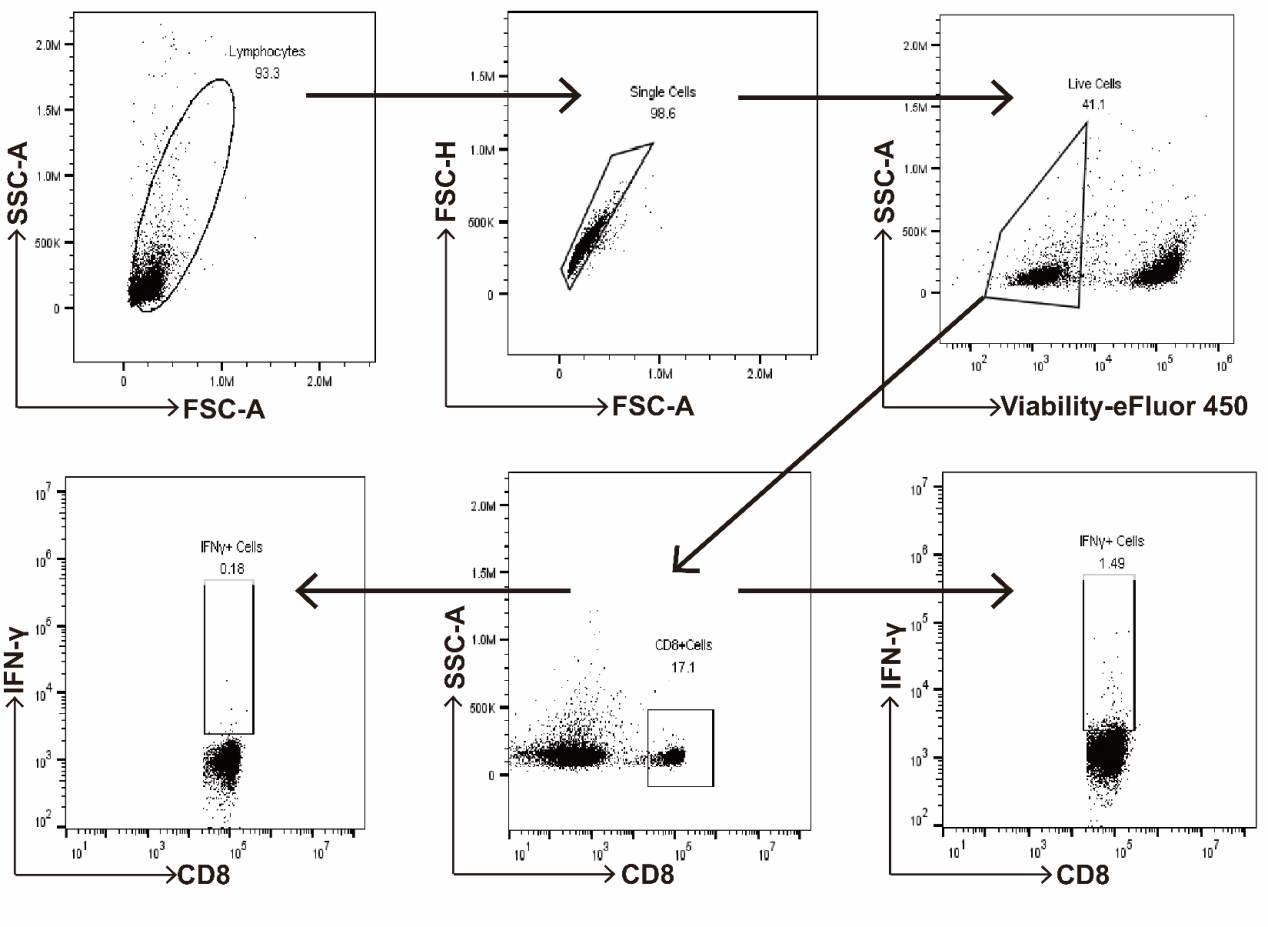


**Figure S4.** Flow cytometric gating strategies for the identification and quantification of IFN‐γ^+^ CD8^+^ T cell subset.


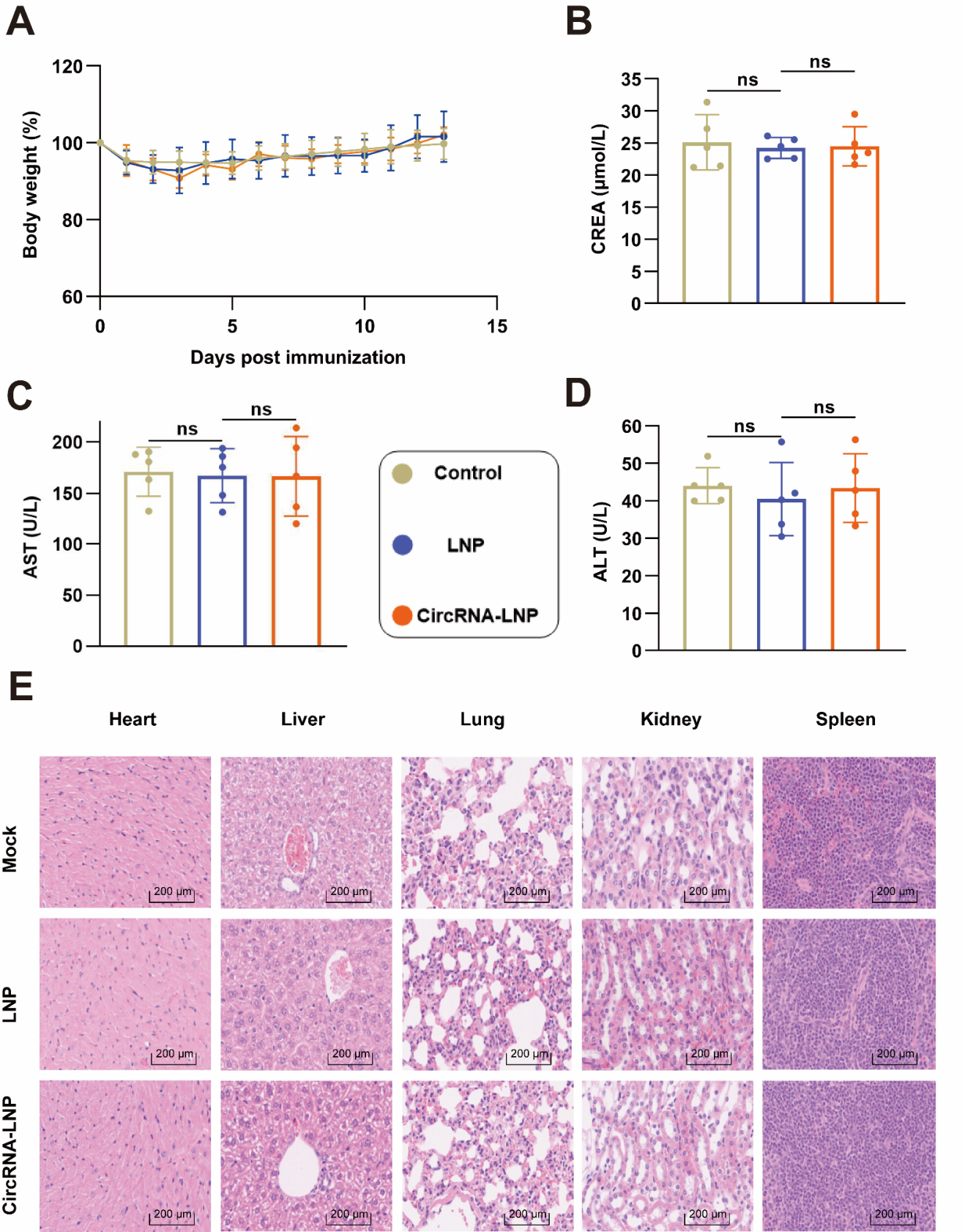


**Figure S5.** **Safety evaluation of the optimized bivalent circRNA vaccine.** (A) The body weight changes of the immunized mice were monitored for 14 consecutive days after vaccination. (B-D) The liver and renal functions were evaluated based on blood biochemical parameters, CREA (B) indicates renal function, while ALT (C) and AST (D) represent liver function, n=5. (E) Hematoxylin-eosin (H&E) stained analysis of various tissues, including the heart, liver, spleen, lung, and kidney collected from immunized mice. The sections shown are representative results from 3 tested mice, n=3.
